# Neuralgia of the Testicle

**Published:** 1873-04

**Authors:** 


*Neuralgia of the Testicle.*

Several years have now elapsed since Dr. Lazarus, of Cnernowitz, reported a number of instances in which spermatorrhœa was accompanied by neuralgia of the testicle. Since then, he has continued to give especial attention to cases of this nature which have come under his notice in hospital and private practice. He finds that in a very limited number of cases only is the entire testicle affected by neuralgic pains; these are commonly limited to the *epididymis*, more particularly the superior portion, together with a part of the *vas deferens*. The left testicle is more frequently affected than the right. The precise pathological changes which take place in the diseased parts have not yet been satisfactorily made out. In a few instances, however, there has been noticed a moderate swelling of the organ, with slight enlargement of the bloodvessels. The predisposing causes of this affection do not differ from those of ordinary neuralgia. It may be the result of idiopathic or traumatic inflammation, or exposure to cold and dampness, or may be induced by the mechanical pressure of some adjacent tumor.

The most common cause, however, of this form of neuralgia is long-continued sexual continence, so that, as would naturally be inferred, the list of this class of sufferers is made up very largely of bachelors. At times, it appears to be a concomitant of approaching impotence, caused here by the engorgement of the bloodvessels and seminal ducts. In such cases, marked relief has been known to follow a natural evacuation of these vessels. In other instances, neuralgia of the testicle makes its appearance in the case of broken down individuals suffering from dyspepsia. Still another cause is the induration which not unfrequently remains behind after a protracted inflammation of the epididymis. In two instances, neuralgia of this organ was found to follow the injection of a strong solution of permanganate of potash and sulphate of copper in the course of a treatment of gonorrhœa. Renal calculi, during their passage along the ureter, may also lead to an attack of this form of neuralgia. In one case seen by Dr. L. the affection was caused by a fall from a lofty scaffolding, by which the lumbar portion of the spinal column received severe injury, resulting in paralysis of the lower extremities and eventual death.

Finally, neuralgia of the testicle is not unfrequently an accompaniment of varicocele. The affair generally begins with a sensation of pain in the upper portion of the epididymis, which continuing without remission, would point to commencing inflammation of that organ, were it not for the absence of the ordinary signs of inflammation, such as redness and swelling. All doubt as to the real nature of the trouble is very soon removed, however, by the change in the severity of the pain, which now comes on in paroxysms of a burning, boring character, accompanied at times by nausea and vomiting. This pain is renewed and aggravated by motion, or whenever any pressure is applied to the scrotum. Cold applications afford no relief whatever to the sufferer; heat, on the other hand, serves to alleviate the pain, particularly when applied in the form of warm baths. This fact will explain why the patient finds himself more comfortable in heated apartments, such as the ball-room or theatre; and also why the paroxysms are less severe in summer than in winter.

Among the agents recommended for the relief of this form of neuralgia, Dr. L. speaks (*Wiener Med. Presse*,) most highly of sulphate of zinc, given as follows:

R. Zinci Sulphatis, gran. tria;
Aquæ Destillatæ, unc. quinque;
Aquæ Laurocerasi, drach. unam;
Syrup. Cort. Aurantium, unc. semis.
M. Dose—Tablespoonful three times daily.

He also advises the daily injection into the posterior wall of the scrotum of a small quantity of the solution of sulphate of zinc, having the strength of one-half grain to the ounce. In obstinate cases it may be necessary to resort to castration.—*The Clinic.*

---

Our neighbors of the *Northwestern Christian Advocate* are responsible for the following "copy" which we give as we swallow our theology, without note or comment:

"We give some statements recorded in the *Gazette Hebdomadaire* taken from *Virchow's Archives*, a medical journal published at Berlin:

"A soldier who had killed the colonel of a regiment in cold blood, and whom the severity of Prussian military discipline would have caused to die a hundred deaths had it been possible, was deliberately handed over to the surgeons, by sentence of court-martial, and was confined in a strong room in the military hospital, entirely in the dark as to the fate which awaited him. He was kept there ready for an emergency which did not fail to occur. A keeper of a beer-cellar in Leipzig, a man resembling, in many respects, the condemned soldier, and who had been seized with acute inflammation of the heart, or rather of its investing membrane, was brought to the hospital to die of that incurable and promptly fatal malady. No sooner had the anticipated death taken place than the dead saloon-keeper was placed on a table by the side of another operating table, on which was the chloroformed but living body of the soldier. Two surgeons, with assistants, proceeded alike in both cases to divide the scalp over the summit of the skull from ear to ear, turn back the divisions, and remove the skull-cap by incisions passing around the skull like a crown. In the soldier, whose carotid arteries had been prepared for compression, these vessels were clamped so as to prevent hemorrhage, and but a few drops of blood were lost during the entire operation. In each the dura-mater was incised, and the hemispheres of the brain were removed by an incision with a sharp, thin-bladed knife, passing above the cerebellum, or a narrow portion of about two inches in diameter called the crura cerebri. The brain of the saloon-keeper, which was sound, the heart disease having left it intact he having been sensible to the last, was transferred to the skull of the soldier, and by an ingenious contrivance, fully detailed in the *Gazette*, the continuity of the arterial and venous tubes was established. The greatest care was taken in securing the natural adaptation of the parts to a fraction of a line, and the skull, having been replaced simply, was held down and in position by the scalp, which was drawn over, and its edges confined by strips of adhesive plaster, and over all was placed a bandage. It was not until several days had passed that the pressure upon the carotid arteries was entirely relieved, although before the skull was replaced the flow of blood in the vessels of the brain was proved to be restored. The chief fear was from the results of inflammation and suppuration, but fortunately neither ensued, and the wounded parts healed kindly. There was from the first no difficulty in feeding the patient, nor was difficulty anticipated, for it is well known that in puppies and kittens in which the entire brain has been removed, sucking and swallowing go on as well as before the operation, and in this case the nerves which preside over deglutition and digestion were far below the point of section. The patient remained in a sound sleep for two weeks, as in a case of apoplexy, the circulation, digestion, and all the vegetative functions of life being uninterrupted. The gradual union of the parts was shown by faint but gradually increasing movements of the limbs, of the jaws, and of the muscles of expression in the face. Speech did not become possible until the close of the third week, and then it was hesitating, stammering, as a child learns. Although it was evident that the patient tried to utter words and sentences, it was very gradually that the power of intelligible articulation returned.

" The *Gazette* contains the report in a tabular form of the increasing voluntary power over the arms and hands, as measured from day to day by the dynamometer, the measurements given in kilogrammes; also the daily temperature of the limbs, as shown by the thermometer in degrees of centigrade; also the measure of returning sensibility of the fingers and lips, as given by an instrument called an æsthesiometer; but I omit these, as your readers will be interested in the main facts only.

" When speech became intelligible it was found that the soldier, as he seemed, had forgotten entirely his military training and discipline; on the other hand he told, at a formal examination, in the presence of a number of witnesses, the prices of all the wines and beers, such as the saloon-keeper had been in the habit of buying and selling, manifesting the unimpaired cerebral activity of the latter. His memory recalled the saloon-keeper's relatives, friends, and customers, whom he called by name. The soldier had been ugly, taciturn, revengeful; he now had the saloon-keeper's frankness, and even garrulity, in spite of his stammering utterance. He was totally blind. Although the nerves of smell and sight had been approximated in the operation, they failed to unite. It was both sad and strange to see and hear the soldier groping in his infirmity of blindness and giving proof of all the patient endurance and goodness of heart, which had made the saloon-keeper deservedly esteemed and prosperous. These are the main facts in the case as far as detailed in the *Archives*, but the subject of experiment presents so many important problems of the relation between blood and brain, of heart power and nervous energy, that we may well be assured that no facts of interest in the changed condition of the culprit will be permitted to escape notice and record. A grave point of discussion is, whether he must still be considered a criminal and suffer execution as a guilty soldier, or shall be pensioned and liberally cared for in his infirmity as a guiltless and much suffering beer-seller. Public sentiment is divided. The Emperor William's professors of metaphysics in the Emperor's universities, say it is clearly a case of Ego and non-Ego, and the people seem willing that the matter should rest there as far as the metaphysical aspects of the question are concerned."

